# A roadmap for breeding orphan leafy vegetable species: a case study of *Gynandropsis gynandra* (Cleomaceae)

**DOI:** 10.1038/s41438-017-0001-2

**Published:** 2018-01-10

**Authors:** E. O. Deedi Sogbohossou, Enoch G. Achigan-Dako, Patrick Maundu, Svein Solberg, Edgar M. S. Deguenon, Rita H. Mumm, Iago Hale, Allen Van Deynze, M. Eric Schranz

**Affiliations:** 1 Biosystematics Group, Wageningen University, Postbus 647 6700AP, Wageningen, The Netherlands; 2 Laboratory of Genetics, Horticulture and Seed Sciences, Faculty of Agronomic Sciences, University of Abomey-Calavi, BP 2549 Abomey-Calavi, Benin; 3 Kenya Resource Center for Indigenous Knowledge (KENRIK), Centre for Biodiversity, National Museums of Kenya, Museum Hill, P.O. Box 40658, Nairobi, 00100 Kenya; 4 World Vegetable Center (AVRDC), P.O. Box 42, Shanhua, Tainan 74199 Taiwan; 5 NGO Hortitechs Developpement, 02 BP 1111 Cotonou, Benin; 6 Department of Crop Sciences, University of Illinois, Urbana-Champaign, IL 61801 USA; 7 Department of Agriculture, Nutrition, and Food Systems, University of New Hampshire, Durham, NH 03824 USA; 8 Department of Plant Sciences, University of California, Davis, CA 95616 USA

## Abstract

Despite an increasing awareness of the potential of “orphan” or unimproved crops to contribute to food security and enhanced livelihoods for farmers, coordinated research agendas to facilitate production and use of orphan crops by local communities are generally lacking. We provide an overview of the current knowledge on leafy vegetables with a focus on *Gynandropsis gynandra*, a highly nutritious species used in Africa and Asia, and highlight general and species-specific guidelines for participatory, genomics-assisted breeding of orphan crops. Key steps in genome-enabled orphan leafy vegetables improvement are identified and discussed in the context of *Gynandropsis gynandra* breeding, including: (1) germplasm collection and management; (2) product target definition and refinement; (3) characterization of the genetic control of key traits; (4) design of the ‘process’ for cultivar development; (5) integration of genomic data to optimize that ‘process’; (6) multi-environmental participatory testing and end-user evaluation; and (7) crop value chain development. The review discusses each step in detail, with emphasis on improving leaf yield, phytonutrient content, organoleptic quality, resistance to biotic and abiotic stresses and post-harvest management.

## Crop breeding: Tending the unattended

Developing the potential of neglected crops can improve food security and economic stability of impoverished farmers. Orphan crops are local, often indigenous, crop species which have been overlooked by research and underutilized as an agricultural resource. Eric Schranz of Wageningen University and his colleagues around the world have reviewed what is known about orphan leafy vegetables and provided guidelines to coordinate research into their breeding and cultivation. The guidelines include increasing awareness of orphan crop potential, collecting and characterizing wild varieties, implementing a breeding program based on modern genetics and genomics, and integrating the needs of key players throughout the process. Orphan leafy vegetables offer an excellent opportunity for small-scale farmers to feed and support themselves, but properly developing this resource requires a concerted effort from researchers, seed companies, policy-makers, farmers, and consumers.

## Introduction

One of the main challenges for agriculture in the coming decades is to meet the nutritional requirements of the nine billion people expected by 2050^1^. World population growth, coupled with the effects of climate variability and increasing competition for water and land resources, makes achieving nutritional security an even more daunting task. While over 5000 plant species are recorded as food plants^2^, <20 species provide most of the world’s food; and three cereals—rice, wheat, and maize—account for ~60% of the calories and ~56% of the protein that humans consume directly from plants^3,4^. The bulk of edible species in the world are therefore non-commodity crops that are mostly overlooked by research and development initiatives. Thus, they are often referred to as orphan, minor, neglected, underutilized, and/or unimproved crops. Orphan crops are also often indigenous, native species or those introduced centuries ago that are still used locally or even regionally, with much untapped potential to increase nutritional security^5,6^. Such species contribute to regional diets, are often adapted to local environmental stresses, and may already be integrated into existing production systems, yet there is little investment to improve their productivity or quality.

Adding value to orphan crops can lead to better livelihoods and improved income generation, especially for smallholder farmers. Such species may also contribute to enhanced climate change mitigation via increased hardiness, reduced external inputs, and subsequent reduction of the carbon footprint of agriculture^4,7,8^. Despite this potential, orphan crops improvement has largely been absent from the global agricultural research agenda, presumably because the relevance of any given orphan crop species is highly geographically and culturally specific. Public agricultural funds are rarely allocated to enable orphan crop research and development, leaving farmers often unsupported in their quest for better use of local agro-biodiversity. Several challenges impede the utilization and conservation strategies of orphan crops, including low productivity, limited variety development, lack of consumer awareness, absence of a value chain, and loss of knowledge. Ongoing efforts in Africa and Asia to overcome such bottlenecks include the documentation of knowledge by the Plant Resources of Tropical Africa (PROTA) and the Plant Resources of South-East Asia (PROSEA) Programmes (prota4u.org; proseanet.org); the germplasm conservation and improvement efforts at the World Vegetable Center; the assembly and definition of genetic diversity of 101 orphan crop genomes and training of plant breeders by the African Orphan Crops Consortium Initiative (AOCC, africanorphancrops.org) and the Alliance for the Green Revolution for Africa (AGRA) to accelerate improvement of neglected and unimproved species of importance for local communities in Africa. How to translate these efforts into tangible breeding outputs for African markets remains an important issue that requires thorough attention. The urgent need to reduce malnutrition and hunger triggers the consideration of orphan leafy vegetables as a viable strategy recommended by the FAO and the WHO^9^ to nourish the overgrowing world population. Strategies adopted to develop orphan leafy vegetables value chains should be aligned with the needs of local populations for access to nutritious and affordable food crops, well adapted to local conditions and available year-round.

This paper serves both as a review of current knowledge and as a roadmap for the genome-enabled development of orphan leafy vegetables. These nutritious, short cycle crops represent the bulk of African orphan crops^10^ and substantially contribute to local communities’ safety nets during food shortage. The demand for these crops is increasing in urban areas of Africa as affordable and available sources of nutrients. Thus, they constitute a significant share of local vegetable markets. The diversity of these species across the continent, including the wide variation in production and consumption patterns, calls for the development of appropriate breeding strategies to meet both farmer and consumer preferences. However, for most of these species, basic knowledge is still lacking related to their reproductive biology, physiology, resistance/tolerance levels to biotic and abiotic stresses, the degree of natural variation, and genetic basis underlying traits of interest. Genomic resources are also lacking for leafy species, which have received less attention than other groups of orphan crops such as legumes^11,12^, grain crops, millets^13,14^, and root and tuber crops^15,16^. Additional considerations are therefore required for breeding of these species to highlight knowledge gaps and direct future efforts.

Throughout this review, the following questions are addressed: Why do we need to improve orphan leafy vegetables? What are the research gaps hindering production and promotion of these species? What would be the key components of a successful breeding program for orphan leafy vegetables, taking advantage of modern advances in genomics? To showcase ways and processes to develop cultivars of useful orphan leafy vegetables for Africa, we used spider plant (*Gynandropsis gynandra* (L.) Briq. syn. *Cleome gynandra* L.) as an example.

## Why breed orphan leafy vegetables?

Orphan leafy vegetables play a significant role in livelihoods, nutrition, and health in marginal areas of Africa. These crops are mostly grown and commercialized by women and contribute to income generation^17–19^. Urban and peri-urban orphan vegetable production employs vulnerable groups, often migrants who came to cities in search of jobs. In Senegal, the contribution of these species to the income of households can be as high as 100%^18,20^. In East Africa, these species are most commonly cultivated and sold in local markets, supermarkets, or green grocery stores, providing income to various stakeholders along the value chain^21^. The average profit margin is estimated to be 30–45% of the selling price^17,19^. For example, *G. gynandra* contributes as much as 15–40% of the total income of some small-scale farmers in Kenya. The price for fresh leaves ranges from 0.40–0.50 USD/kg during the rainy season but can double in value during the dry season when vegetables are less readily available^19,21^. A survey conducted on 861 indigenous vegetables retailers sampled in seven African countries revealed an annual turnover of 5.5 million USD. Weinberger and Pichop estimated that African indigenous vegetables market is worth billions of USD across Sub-Saharan Africa^19^.

Orphan leafy vegetables can provide affordable and locally available sources of nutrients including vitamins, minerals, and protein^22–29^. Beyond their nutritional value, orphan leafy vegetables are also used as medicinal plants in various communities^30–33^. For instance, various parts of *G. gynandra* are used to strengthen the immune systems of women and children, as well as to cure wounds, diverse inflammations, digestive disorders, epileptic fits, and malaria^34–36^. Its high vitamin (e.g. provitamin A, vitamin C) and micronutrient content (e.g., iron) makes the species particularly important in expecting and lactating mothers as well as in child development. Pharmacological studies revealed high concentrations of glucosinolates, flavonoids, tannins, iridoids, and other phytochemicals in the leaves^37,38^, conferring to the plant proven antifungal, antibacterial, antiviral, anticarcinogenic, analgesic, febrifuge, and anti-inflammatory properties^39,40^.

Beside their great potential as both food and medicine, local landraces of orphan leafy vegetables are an asset to cope with climate variability. They are resistant to adverse environmental factors and can be easily grown in drought-prone areas with low rainfall^41^. However, attempts to breed more productive cultivars have been limited so far despite many features which make these species conducive to genetic improvement. Most of these species have a short cycle from 3 to 5 months and are predominantly self- or out-crossing with a certain rate of out-pollination or self-pollination, which makes them amenable to different breeding strategies^31,42^. For example, *G. gynandra* has a short life cycle of 3–4 months, with plants tending to flower very early, within 4–6 weeks from planting. The species also shows substantial variation in reproductive characteristics relevant to domestication and crop improvement. Flowering is gradual, starting with the terminal shoot and followed by the axillary shoots, and may last for more than two months^43^. The species is both self-pollinated and cross-pollinated^43^, where cross-pollination is facilitated by wind or insects (e.g., honeybees, thrips and butterflies)^43,44^. Two types of flowers are commonly observed in *G. gynandra*: (1) a staminate type consisting of a residual ovary devoid of ovules, which can contribute to cross-pollination; and (2) a hermaphroditic type consisting of a functional ovary and stamens, which permit self-pollination^44,45^. Under stress, individuals bearing flowers with infertile reduced stamens were also observed. The species is self-compatible and facultative autogamous, with allogamy occurring occasionally^44^. Such characteristics are advantageous for the species, as they allow fruit set whether or not pollinators are available and additionally offer flexibility in breeding methods that can be applied to improve *G. gynandra*.

During the last 30 years, the World Vegetable Centre released 13 cultivars of orphan leafy vegetables obtained by single seed descent or mass selection in Tanzania, Uganda, Kenya and Mali. These include five African nightshades (*Solanum scabrum*), five amaranths (*Amaranthus* spp.), two Ethiopian mustards (*Brassica carinata*) and one jute mallow (*Corchorus olitorius*)^46,47^. Leveraging genomics-assisted breeding strategies to sustain current breeding efforts in orphan leafy vegetables in a concerted manner would therefore be beneficial for the development of commercial value chains for these species.

## Developing breeding programs for orphan leafy vegetables

Developing breeding programs for orphan leafy vegetables begins with cultivar development based on consumer preferences and adequate adaptation to various ecological conditions, with precise product targets being dictated by individual market regions. Typically, smallholder farmers seek full-season varieties with high leaf yield, resistance to diseases and pests, and abiotic stress resistance (e.g., drought, heat, and salinity tolerance). Retailers and consumers seek good appearance, long shelf-life, superior taste and aroma, high nutritional value, and affordability^47^. As both growers’ and consumers’ preferences are important in defining breeding objectives and product targets, they should be investigated at an early stage of the breeding program to guide germplasm collection and characterization strategies, and then prioritized in later stages once information on genetic diversity and breeding constraints become available. Cultivar development with diverse stakeholder participation (including farmers, retailers, and consumers) can enable breeders to create varieties with desired traits, reduced adoption bottlenecks, and broad acceptability^48^. Furthermore, genomic tools have the potential to accelerate the entire cultivar development process, provided the product target is stakeholder-driven and well defined.

**Fig. 1:**
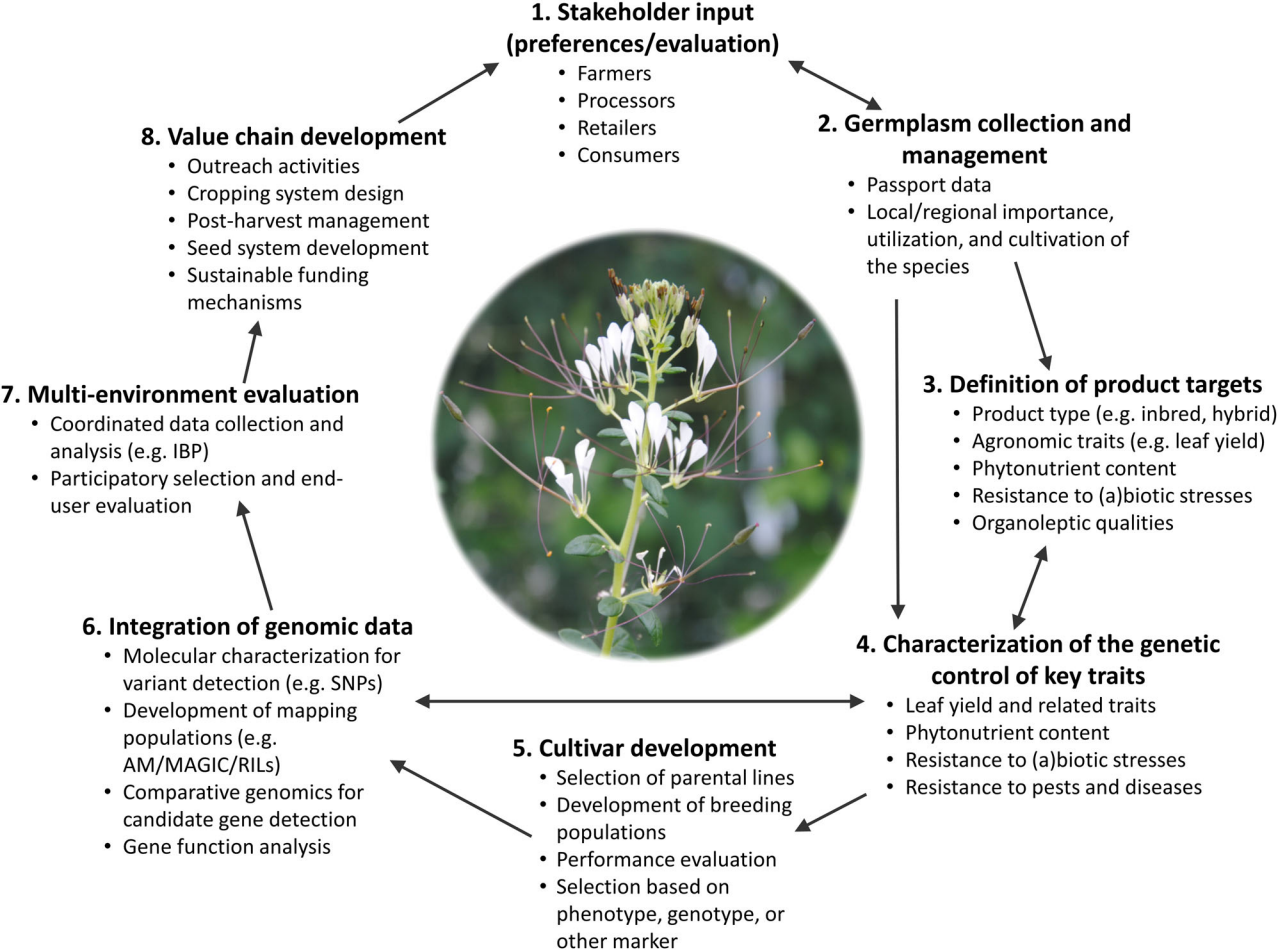
Schematic of an integrated breeding program for orphan leafy vegetables

In the next section, we propose a breeding framework based on a multi-disciplinary approach which takes advantage of modern advances in genomics and breeding to guide concerted, inclusive efforts by researchers to ensure improved nutritional outcomes for consumers. The following steps are identified as milestones for achieving a successful improvement program for orphan leafy vegetables: (1) germplasm assembly, characterization, and management; (2) definition of product targets, (3) characterization of the genetic control of key traits; (4) design of the process of cultivar development; (5) integration of genomics data to optimize that process; (6) multi-environment participatory trials and end-user evaluation; and (7) crop value chain development (Fig. 1).

### Germplasm assembly, characterization, and management

Knowledge of the taxonomy, distribution, and ecology of a crop species is a prerequisite for proper germplasm assembly. Such information is available for most orphan leafy vegetables^31^ and should be sought for in species with such knowledge gaps.

With information of the taxonomy and distribution of the species, regions can be prioritized and germplasm collection strategies developed. All collections should be accompanied by detailed passport data, including accurate geo-referencing, habitat characterization, and sampling methods, in order to facilitate the downstream interpretation of genetic data of genebank accessions^49^. Sampling strategies must be defined that ensure an optimal coverage of habitats relevant to key traits of interest^50,51^. Ethnobotanical data related to the importance, cultivation, and utilization of the species should also be recorded at each collection site, as such data help bring function-related structure to the collection of genetic diversity.

Orphan leafy vegetables are usually conserved ex situ. Ex situ germplasm collections of some orphan leafy vegetables already exist in local, regional, and international gene banks but need to be expanded. For example, 295 accessions of *G. gynandra* are currently maintained at the World Vegetable Center, including 112 accessions from Eastern and Southern Africa and 183 from Asia (http://seed.worldveg.org/). Thirty-one accessions from Southern Africa are held within the National Plant Germplasm System of the USDA (http://www.ars-grin.gov/). Collections are also maintained in Botswana, Kenya, Namibia, Tanzania, Zambia, and Zimbabwe^52^. More recently, we assembled accessions from Benin, Togo, Ghana, Niger, Burkina Faso, and Kenya, resulting in a collection of 164 accessions from West Africa and 52 from Kenya. This new collection is currently maintained at both UAC and KENRIK and will soon be integrated into larger gene banks, such as that at the World Vegetable Center. In building this important new germplasm collection from West Africa and Kenya to support *G. gynandra* breeding programs, a standardized collection form was developed for the species (Supplementary file 1). Leveraging this initial work, future collection missions in South and Central America as well as Australia could help enhance the global collection of *G. gynandra* diversity. Available germplasm of orphan leafy vegetables must be continually enriched with purified lines, cultivars developed by research institutions, collections from farmers, as well as plant material collected from the wild. Conservation strategies to maintain genetic diversity depend on the mode of reproduction and fecundity of materials. Self-pollinated accessions are maintained as a single plants when they are pure breeding lines but also as populations in the case of landraces and diverse materials. Cross-pollinated species are mainly maintained as populations paying attention to inbreeding depression and genetic drift. Collections should be performed and distributed in accordance with the national and international germplasm exchange policies such as the International Treaty on Plant Genetic Resources for Food and Agriculture.

Germination and dormancy can be an important constraint for the successful conservation and utilization in orphan leafy vegetables germplasm as is the case for *G. gynandra* and *S. scabrum*^20^. In *G. gynandra*, germination percentages between 25–65% were reported for seeds collected from research organizations in Kenya, compared with 15% from farmers’ fields^20^; and light exposure has been shown to inhibit seed germination^53^. Furthermore, a variable after-ripening period, ranging from 3 months^54^ to 2 years^55^, has been shown to increase the germination rate up to 90%. Various pre-germination treatments, including imbibition with potassium sulfate (K_2_SO_4_) or gibberellin (GA_3_)_,_ and germination at 30 °C in the darkness, also improved germination rates^53,55–57^. Protocols for proper seed conservation and efficient germination, without need for an extended after-ripening period, are needed to shorten the breeding cycle of *G. gynandra* and avoid the unintended erosion of ex situ genetic diversity due to selection against poor germination.

Linking genotypic and phenotypic variation to socio-ecological context is one means of gaining insight into the adaptation processes under different climatic conditions as well as the impacts of domestication on the species^58^. The long-term perspective should combine phenotypic and genotypic characterization data for the development of core collections to be shared among gene banks for regionally specific breeding goals^58^. However, the development of molecular markers for characterization of the genetic diversity in orphan leafy vegetables should be pursued. To date, mainly second-generation molecular markers including random amplification of polymorphic DNA (RAPD), amplified fragment length polymorphisms (AFLPs), and microsatellite or simple sequence repeats (SSR) have been used to investigate genetic diversity in orphan leafy vegetable species^59^ such as *Corchorus olitorius*^60–62^, *Brassica carinata*^63,64^, *Solanum scabrum*^65^, and *Gynandropsis gynandra*^66^. Although these preliminary studies gave base information, the markers used are not economical for breeding. Discovery of single-nucleotide polymorphisms (SNPs), insertions/deletions (indels), and copy number variation is yet to be exploited for most orphan leafy vegetables, although some exceptions have been reported for genetic characterization of *Vigna unguiculata*^67^, *Brassica carinata*^68^, and *Amaranthus* spp.^69,70^ which are also valued as pulse, oilseed and pseudo-cereal, respectively.

### Definition of breeding goals and objectives

The type of cultivar to be developed is a critical decision, based on the reproductive system of the crop, the presence of hybrid vigor and access to male sterility systems, as well as the seed distribution systems either already in place or to be developed in the market region. The aim is to develop a uniform, reproducible product that stably expresses all the target traits of interest and can be produced at low cost. Such a product can take the form of a pure line variety, an open-pollinated variety, a hybrid (e.g., single cross, three-way cross, or double cross), or, in some cases, a clonally propagated cultivar. In Africa, where farmers may choose to save seed rather than purchase seed each year, a pure line variety may be preferred, if reproductive mechanisms allow. Although, maintaining performance and purity of cultivars through well-managed seed production is essential to gain the value of improved cultivars.

As is true of most crops, important target traits for orphan leafy vegetables include increased yield, higher nutritional content, resistance to pests, and tolerance to relevant stresses such as heat, drought and salinity. In addition, the maturity of any improved cultivar must be aligned with individual market regions; and consumer preferences must be honored. For example, our recent investigations of farmers’ preferences in Benin and Kenya revealed distinct regional flavor preferences for *G. gynandra*: no bitterness in East Africa and slight bitterness and spiciness with strong aroma in West Africa. Baseline data, together with ethnographical studies, are therefore essential in identifying not only promising breeding populations but also the environments and methods required for the meaningful evaluation of target traits.

### Characterization of the genetic control of key traits

Although some information on farmer and consumer preferences is available, little is known about the genetic control of key traits of interest; yet such knowledge is critical for designing a breeding program. Factors such as the number of genes controlling trait expression, the type of gene action involved (e.g., additive, dominant, and epistastic), the magnitude of genetic and phenotypic variances, the possible interactions with the environment, and the heritability of key traits can influence the design of the “process” by which improved cultivars will be developed. That such information is scarcely available for orphan crops implies that knowledge of genetic variances (additive, dominant, and epistatic) and heritability of traits of interest should be generated. Genetic variances are commonly estimated using procedures such as diallel, nested, and factorial designs; and the phenotypic evaluation of traits must take into account environmental and market specificity. Detailed descriptions of common estimation procedures are provided by Dudley and Moll (1969)^71^ and Fehr (1987)^72^.

Furthermore, other characteristics correlated with key traits may be utilized to devise more efficient and cost-effective breeding strategies. For example, simply measured traits of an individual plant, such as height or total leaf number, may serve as reliable proxy metrics for a key system-level trait like leaf yield per area. Typically, the product target includes a balance of traits related to yield potential (e.g., resource partitioning and traits to protect that potential including defensive traits such as pest resistance and stress tolerance). It is important to understand as much as possible about the genetic architecture of target traits from the start of a breeding program. For example, in the very early stages of a breeding program, association mapping using natural populations can be performed to explore population structure and the genetic control of the target traits, once phenotypic and genotypic data become available^73^. Such preliminary results can guide subsequent efforts of molecular dissection of complex key traits; and more will be learned as strategic mapping populations are developed and genomic technologies are employed to map genes and estimate the magnitudes of their effects^74^.

The phenotypic characterization of selected traits in orphan leafy vegetables should be achieved using a set of standardized protocols, developed and shared among research institutes in an effort to facilitate meaningful data comparison across environments. Morphological characterization data is available in some species including *Amaranthus* spp.^75–77^, *G. gynandra*^78^, *S. scabrum*^79^, *V. unguiculata*^80^, and *C. olitorius*^81,82^. A standard list of morphological descriptors has been developed for *G. gynandra* by researchers at the World Vegetable Center and revised by the Cleome Consortium (Supplementary File 2). Gene banks and other institutions working on *G. gynandra* are encouraged to use and participate in the ongoing refinement of such a list of phenotypic descriptors. Descriptors used by the World Vegetable Center for some genera of importance including *Basella*, *Celosia*, *Cleome* (*Gynandropsis*), *Corchorus*, *Ocimum*, *Solanum*, *Talinum*, and *Vigna* are available (http://seed.worldveg.org/download) and could be used for large-scale characterization of target species.

Affordable techniques for high-throughput phenotyping^83,84^ should also be considered, especially as such methods are developed for other species with similar plant architecture. For example, the phenomics software Tomato Analyzer (https://www.jove.com/video/1856/tomato-analyzer-useful-software-application-to-collect-accurate) developed for tomato was used to characterize other *Solanum* species including *S. macrocarpon*^85^. These methods are useful if objective data can be collected efficiently with high correlation to targeted phenotype.

#### Leaf yield-related traits

**Fig. 2:**
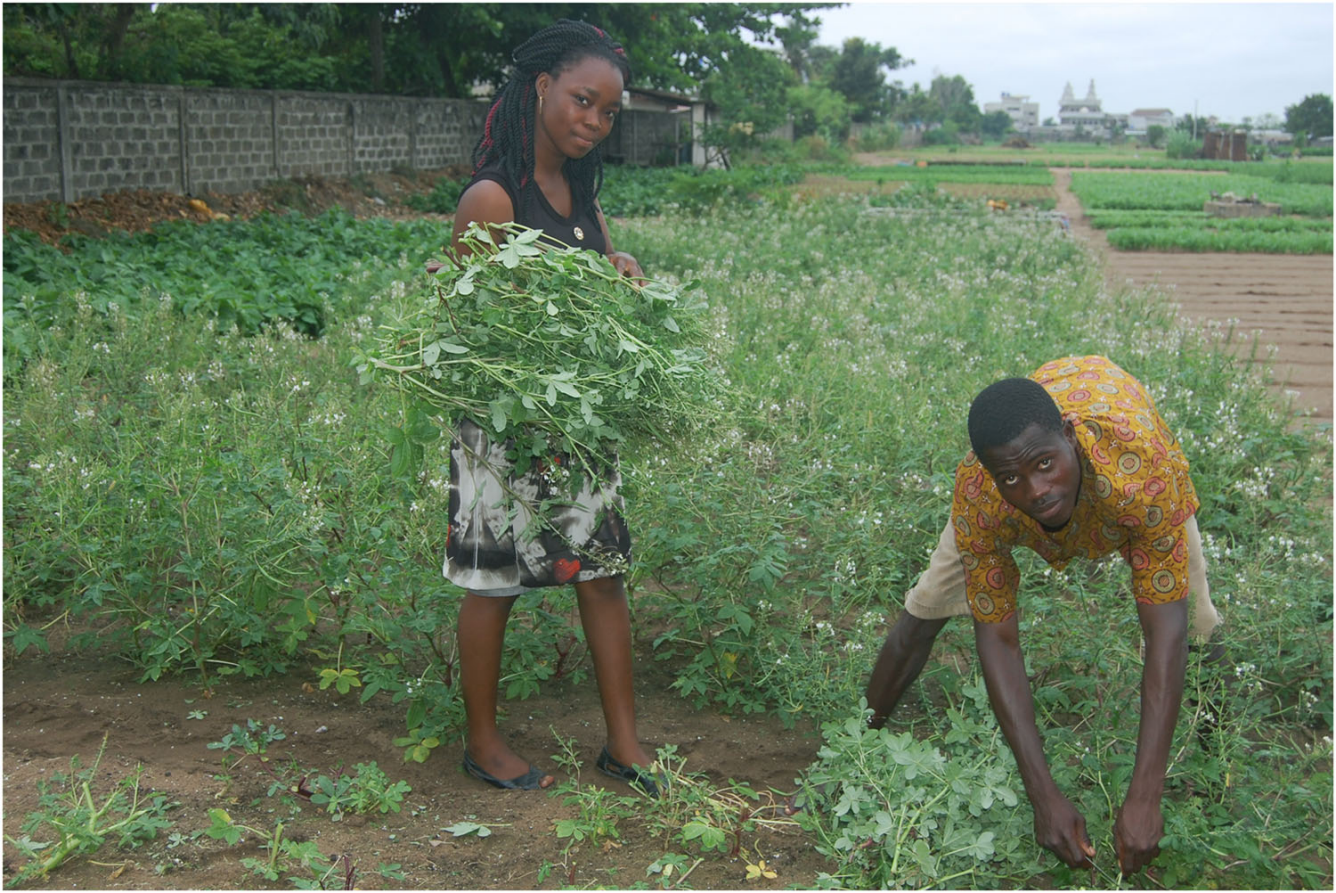
Farmers harvesting *Gynandropsis gynandra* in a peri-urban garden (Benin)

Leaf yield is the first and foremost target trait in leafy vegetables breeding. Breeding leafy vegetables requires proper characterization and strong correlations between phenotypic traits and leaf yield. Such studies were undertaken for some species including *G. gynandra*^86^, *A. tricolor*^87,88^, and *B. carinata* though not extensively. In *G. gynandra*, Omondi^86^ estimated low heritabilities for leaf yield and yield-related traits, including plant height, number of leaves, leaf length, and leaf width, but a high heritability for days to flowering, a measure of maturity. Chweya and Mnzava^22^ reported a negative correlation between days to flowering and leaf dry weight in the species, indicating that leaf yield in *G. gynandra* may be maximized with full-season varieties. Further investigations including several genotypes and locations are required to validate these observations and assess other important traits. High heritabilities and genetic advances were estimated for leaf yield and positively correlated traits such as plant height, number of leaves, and stem diameter in *A. tricolor*.^87,89^ Therefore, leaf yield in *A. tricolor* could be significantly improved through direct selection for these traits. Further studies are required to document and better understand farmers’ preferences in this respect. For instance, farmers who adopt a multiple harvesting strategy could be interested in plants developing many branches in a short amount of time, while late-flowering plants may not have this feature. Indeed, Chweya and Mnzava^22^ reported that good moisture supply at the early stages of plant growth promotes fast vegetative growth with reduced branching, while plant stress promotes early branching. Harvest index should also be considered, as late-maturing types may exhibit a high total biomass while the proportion of edible biomass may be low. The ability to regenerate after cuttings and the cost-effective number of cuttings should also be considered when selecting for leaf yield in regions where farmers adopt multiple cuttings (Fig. 2). For example in Kenya, where whole plants of *G. gynandra* are uprooted 4–5 weeks after sowing, emphasis should be put on fast vegetative growth rather than cutting ability. Finally, to date, there is little information available on the potential for hybrid vigor in leafy vegetables. A diallel crossing scheme could be used to develop materials to evaluate general and specific combining abilities, which could be exploited in cultivar improvement.

#### Phytonutrient content and consumer preferences

The significant impact of micronutrient deficiencies on human health, especially in developing countries, is gaining recognition^47^. Breeding for high-yielding varieties of orphan leafy vegetables that are both nutrient-rich and low in anti-nutritional factors may be an effective means of achieving biofortification, while simultaneously contributing to diet diversification and rural livelihoods. In a collection of one hundred accessions of *G. gynandra* from West Africa, East Africa, and Asia, nutritional content is being assessed by the Cleome Consortium using a multi-platform metabolomics approach able to provide a comprehensive characterization of qualitative and quantitative variation in a wide range of metabolites. Analytical platforms include, but are not limited to: high performance liquid chromatography-photodiode array-fluorescence for apolar compounds (e.g., carotenoids, tocopherols, and ascorbic acid); liquid chromatography–mass spectrometry for semi-polar compounds (e.g., alkaloids, glucosinolates, flavonoids, and gallotannins); and gas chromatography–mass spectrometry for volatile compounds (e.g., amino acid derivatives, fatty acid derivatives, and terpenes). The results of such untargeted metabolomics methods may be combined with proximate and mineral analyses to finely characterize nutrient content variation in germplasm collections and guide selection. Results from such analyses will therefore provide rational nutritional targets in *G. gynandra* as well as a basis for meeting them. With proper passport data, intraspecific variation in metabolic profiles may also be linked with cultural and/or geographical information. Moreover, the discovery and identification of metabolites with health-promoting properties could motivate further pharmacological studies on the species and provide incentive for utilization of *G. gynandra* as a nutraceutical food. While there is available information on the nutritional value of orphan leafy vegetables^24,27^, such comprehensive analyses on natural variation in nutrient contents on wide germplasm collections are scarce and should be considered.

To help ensure adoption of improved varieties, the development of nutritional traits and nutrient-rich lines must be pursued with full knowledge of consumers’ preferences. It is therefore essential that country- and/or region-wide organoleptic tests be performed, taking advantage wherever possible of existing partnerships and prioritizing areas of extant demand for target species. For example, the bitterness of *G. gynandra* is not desired in eastern African countries, as evidenced by the various cooking methods used to attenuate it. In Botswana, the leaves are initially blanched in water and the water discarded and replaced with a fresh supply^90^. In Kenya, milk is added to the leaves in a pot and left overnight to improve the taste. Leaves are also mixed with those of other species, including *Amaranthus* spp., *Solanum* spp., *Basella alba*, and *Brassica carinata*^91^. In contrast, in West Africa and especially in Benin, bitter taste is more tolerated and even appreciated. In this region, bitter leaf (*Gymnanthemum amygdalinum*) is a popular vegetable and hence the bitterness in *G. gynandra* is not perceived as a negative trait. Such regional differences should be taken into account in breeding programs, especially given the objective of actively promoting and increasing the use of orphan crops. For *G. gynandra*, organoleptic tests may be conducted in West Africa and East Africa based on standard criteria selected with trained tasting panels (e.g. bitterness, spiciness, odor, texture). For instance, established correlations between bitterness or odor and specific metabolites could allow the early selection of *G. gynandra* lines with preferred taste profiles.

Cooking methods have a significant impact on the realized phytochemical content and antioxidant capacity of vegetables^92,93^. Across sub-Saharan Africa, leafy vegetables are usually boiled^52^, blanched, or made into small balls and sun-dried for preservation^90^. The differential effects of these and other cooking practices on nutrient content is not well studied in orphan leafy vegetables. Given the diversity of cooking methods used, it is important to assess the impact of common preparation methods on the bioavailability of specific phytonutrients and total antioxidant capacity in order to identify and recommend best cooking practices. Wide dissemination of results and open dialogue with consumers will be needed to understand and influence changes in traditional cooking practices while preserving culinary diversity. Progress in traits associated with phytonutrient content and flavor will also benefit from improved knowledge of their underlying genetic control, their relevant metabolic pathways, and the physiological processes involved. There is an opportunity to leverage comparative genomics between orphan leafy vegetables and well-studied relatives as well as metabolomics strategies toward this end.

#### Resistance to biotic stresses

Orphan crops are generally well adapted to their environment and some species have developed chemical defences against specific pests. For instance, methanol extracts and volatile emissions of aerial parts of *Gynandropsis gynandra* have been shown to have a strong acaricidal effect, especially on the two-spotted spider mite *Tetranychus urticae*^94,95^ as well as on both *Rhipicephalus appendiculatus* and *Amblyomma variegatum*, two livestock ticks occurring in Africa^96,97^. The use of *G. gynandra* as a companion crop in plots of snap bean (*Phaseolus vulgaris*) significantly reduced the incidence of thrip species *Megalurothrips* and *Frankliniella occidentalis*^98^. Volatile compounds with significant repellent activity include aldehydes, terpenes, and isothiocyanates^95,97^, the latter being breakdown products of glucosinolates which occur after foliar disruption^99^.

Perhaps unsurprising, given the close relationship between *G. gynandra* and the Brassicaceae, most of the pests reported for the species also cause damage to cruciferous crops. For example, *Bagrada hilaris*^100^ and *Phyllotreta* spp.^101^ are serious economic pests of *Brassica* species, and such invasive species can be expected to have a stronger incidence in vegetable production systems where both *G. gynandra* and cruciferous crops are grown. Other pests commonly affecting orphan leafy vegetables production include caterpillars (e.g., *Helicoverpa armigera*, *Plutella xylostella*, and* Spodoptera* spp.,) nematodes (*Meloidogyne* spp.), thrips, aphids (*Aphis* spp.), and whitefly (*Bemisia tabaci*). Judicious crop associations by leafy vegetable growers can therefore reduce the incidence of some pests and diseases.

Several insect pests of *Brassica* spp. are reported to be preferentially attracted to genotypes with specific metabolic profiles, related to levels of glucosinolates, amino-acids, and sugars^102,103^, a result indicating both opportunities and difficult trade-offs. Indeed, innate chemical defences may be enhanced by orphan leafy vegetable breeders via direct selection under insect pressure or indirectly via metabolomics analyses. In either case, the factors affecting expression of insect resistance must be taken into account^104^. Development of pest resistance must be prioritized on a regional basis.

With sufficient information on economically important diseases in orphan leafy vegetables and prioritization in terms of impact, effective field and greenhouse screening, and laboratory techniques can be developed for identifying tolerant and resistant genotypes. To the extent that such pests and pathogens are shared with more studied crops (e.g., economic *Brassica* spp.), such methods may already be well established. As with insect pests, resistance to pathogens is often associated with specific metabolites in plants. Thus metabolomics approaches may also be useful in detecting resistance-related compounds, particularly for use as biomarkers for selection. In general, morphological and molecular differentiation of pathogenic races and biotypes is a prerequisite for the efficient breeding of durable forms of resistance; and breeders must remain vigilant about the potential impacts of resistance to specific diseases on desired agronomic traits. Strategies for biotic stresses should integrate both management and breeding technologies.

#### Resistance to abiotic stresses

In the tropics, high-temperature conditions are often prevalent during the growing season and, with a changing climate, crops in such areas will be subjected to increased temperature stress during developmental and productive phenostages^105^. Phenotyping for drought and heat tolerance under controlled conditions can be used to pre-screen lines for further verification in the field. Even though field experiments are subject to variation, it is possible to closely monitor environmental parameters or design semi-controlled conditions using specific methods (e.g., rain shelters, irrigation, and enclosures)^106–108^. The developmental stages at which these stresses occur, their duration, and their severity are also key factors for tolerance/resistance evaluation.

Beside yield evaluation under abiotic stresses, an understanding of the physiological processes underlying tolerance of abiotic stresses is needed to determine the physiological and morphological traits to include in selection criteria. For example, variation across genotypes in water uptake, photosynthetic efficiency, and water use efficiency traits like leaf conductance, photosynthetic assimilation rate, chlorophyll content, leaf thickness, leaf nitrogen content, and stable carbon isotope ratio could be investigated, particularly as they relate to leaf yield^109,110^. Leaf relative water content, wilt, and differential plant growth following drought stress have been suggested as indicators of water stress in lettuce screening for drought tolerance^111^; and such parameters could be considered to develop screening methods tailored to each vegetable species. C_4_ species such as *G. gynandra*, *A. cruentus* and *A. tricolor* would develop different drought escape mechanisms compared with C_3_ species. The available information on the genetic control of drought and heat tolerance in well-studied sister species, including *A. thaliana*^112–114^, *Brassica* spp.^115,116^ (e.g., *Brassica rapa*, *B. oleracea*, *B. napus*, *B. juncea*, and *B. nigra*) for *G. gynandra* or *Chenopodium quinoa*^117,118^ for *Amaranthus* spp., should be used to facilitate the genetic characterization of these traits.

### Design of the process of cultivar development

**Fig. 3:**
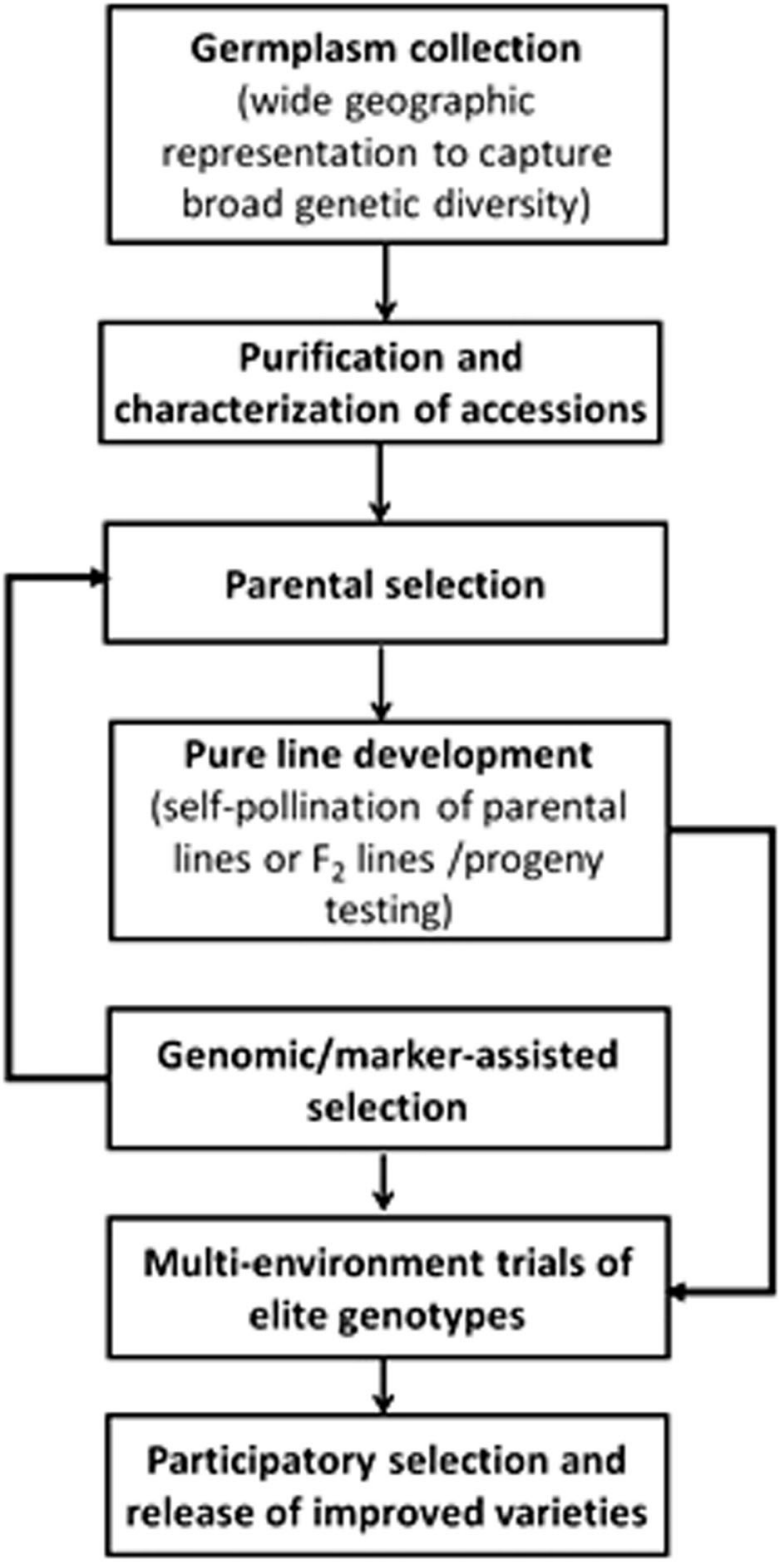
A breeding pipeline for the development of pure line varieties of *Gynandropsis gynandra*

Thorough knowledge about the reproductive biology of the targeted species will be a prerequisite for cultivar development. For predominantly self-pollinating species with some amount of out-crossing, one sensible product target would be pure line varieties (Fig. 3). Controlled crosses can be performed via manual emasculation and out-crossing during breeding can be controlled by covering flowers. Later, varieties can be scaled up for seed distribution in isolated plantings to prevent out-crossing. If evidence is found for hybrid vigor in terms of leaf yield, hybrid cultivars could be pursued as a means to significantly increase leaf yield in the short term. This can be specifically pursued for species like *G. gynandra* and *Solanum* spp. which are both self-crossing and out-crossing. However, as mentioned before, for such a strategy to be commercially viable, seed production and distribution systems must be able to support hybrid seed production. Very likely, some mode of male sterility would need to be developed to facilitate hybrid seed production.

Once the form of the product has been decided (e.g., pure lines vs. hybrids), the critical elements of a breeding program focus on the choice of parents and the evaluation of progeny vis-a-vis well-defined trait targets. Pairs of lines that perform well for all of the key traits (high phenotypic value) yet offer diverse favorable alleles (high genotypic variance) represent ideal parental combinations for crosses. Often referred to as a ‘good-by-good’ cross, such an ideal situation is rarely possible when breeding efforts are first initiated in a crop species. Instead, potential sources of favorable alleles for all the key traits of interest in available germplasm (e.g., leaf yield, time to maturity, resistance to pests and diseases, tolerance to heat and drought) should be identified and a plan for efficiently combining them set out. To begin, a testing system to evaluate the progeny of breeding crosses should be devised to accurately and precisely estimate the heritiabilities of and correlations among the traits in the refined product target, as well as to identify any related traits that could be a basis of selection toward the product target.

The choice of parents will be based on the results of germplasm characterization and the progeny selected based on performance for key traits will be tested at multiple locations representative of the range of agro-climatic conditions under which the final product will be cultivated (see Multi-environment testing and end-user evaluation section for further details). Some orphan leafy vegetables (e.g., *G. gynandra*, *S. macrocarpum*, *Amaranthus* spp., and *C. olitorius*) are fast-growing plants and the whole production cycle (seed to seed) takes from 4 to 6 months, thus allowing two to three selection cycles per year.

Because product targets are never about a single trait, an approach to multi-trait selection must be determined. Such a strategy must take into account the relative importance of the traits for end-users, the nature of those traits (qualitative vs. quantitative), the complexity of their genetic control (e.g., additive, dominant, epistatic, additive x dominant, etc.), their degree of heritability (low vs. high), their correlations to one another, and the selection intensity imposed by the breeder. One efficient means of breeding species of interest for multiple traits (e.g., maturity, leaf yield, and vitamin content) could involve the use of selection indices, particularly if significant negative correlations are found among target traits. To maximize gain via multiple selection cycles per year, it is likely that some selection will need to be done in off-seasons, when phenotypic data may be less reliable. In such cases, marker-assisted recurrent selection using markers with significant effects^119^ or genomic selection^120,121^ with dense, genome-wide sets of markers could be pursued. In the latter case, training populations could be developed based on available association panels; and indeed, genomic selection methods have been used in other crops for accurate phenotypic prediction of a wide range of traits^122–124^. Such genotype-based approaches will increase in their efficacy as deeper knowledge about target trait-related gene function and variation is attained. When dealing with specifically known, single genes, marker-assisted backcrossing could be explored for cultivar development^125–127^. Such an approach seems particularly promising for introgressing disease resistance genes in species of interest and pyramiding them in high-yielding, nutrient-rich cultivars.

### Integration of genomic data

Molecular markers associated with traits or as a tool for whole genome selection are a valuable asset for efficient breeding. Key genomic resources available in *G. gynandra* include a draft reference genome (Schranz et al., unpublished) and a transcriptome atlas of the species^128^. Building upon these research efforts will provide opportunities for increasingly detailed analysis of genetic diversity within the species as well as accelerated trait development in breeding programs.

To facilitate the development of a dense, genome-wide set of molecular markers, 100 diverse *G. gynandra* accessions are being sequenced by the AOCC; and their sequence data will be aligned to the reference genome for global variant calling. Other orphan leafy vegetables on the agenda of AOCC include *Amaranthus blitum, A. cruentus, A. tricolor, Basella alba, Brassica carinata, Celosia argentea, Corchorus olitorius, Crassocephalum rubens, Moringa oleifera, Solanum scabrum*, and *Talinum fruticosum*. On the basis of both genotypic and phenotypic data, divergent parents can be identified and Recombinant Inbred Lines (RIL) populations developed to generate genetic linkage maps, enable QTL analyses, and highlight genomic regions of particular interest to breeding programs. They also enable marker-assisted backcrossing programs for foreground selection of traits and background selection for quick recovery of the recurrent parent genotype. In addition to bi-parental populations and association mapping panels, Multi-parent Advanced Generation Inter-cross (MAGIC) lines could also be used to dissect traits of interest^129,130^. The multiple cycles of inter-crossing among multiple founder lines in MAGIC populations give greater opportunities for recombination, permitting both greater precision in QTL location^131^ and an increased probability of favorable combinations of alleles from the multiple parents. MAGIC populations have been successfully used for QTL mapping in durum wheat^132^, barley^133^, and rice^134^, to name a few. As developing MAGIC lines is time and resource intensive, this approach could be used to complement classical linkage and genome-wide association mapping.

Genotyping services such as DNA extraction and Kompetitive Allele Specific PCR (KASP) genotyping for SNPs and insertions/deletions (indels) are provided for AOCC-designated orphan crops by the LGC Group (www.lgcgroup.com), a member of the AOCC. For orphan crops with no reference genome, recent whole genome analyses and *de novo* SNP calling pipelines like GBS-SNP-CROP^135^ present highly cost-effective alternatives for immediate implementation. Services for such whole genome analyses are available through the Biosciences East Central Africa (BecA) hub in Nairobi. In the case of *G. gynandra*, the draft reference genome enables variant calling and genotypic characterization via a number of open-source pipelines and complementary bioinformatics tools.

In terms of genomic analysis, including functional characterization, advantage should be taken of the significant synteny among orphan species and well-studied crops. Genomics-assisted breeding in *G. gynandra* and *B. carinata* could tap into available information on *Brassica* spp., and *A. thaliana* genomes^136,137^, *S. macrocarpon*, *S. aethiopium*, and *S. scabrum* on *S. lycopersicum* and *S. tuberosum* genomes; vegetable amaranths (*A. blitum*, *A. cruentus*, *A. tricolor*, and *A. dubius*) on the recently published *A. hypochondriacus*^138^ and *Chenopodium quinoa* genomes^139^. Exploiting the available comparative data on physiology, genetics, and “omics” in well-studied crops is an attractive avenue for candidate gene identification in orphan leafy vegetables. In addition, reverse genetics approaches such as Targeting Induced Local Lesions In Genomes (TILLING) can be used as a high-throughput approach for functional genomics based on well characterized genes in closely-related species. Gene editing techniques could also be utilized if needed.

### Multi-environment testing and end-user evaluation

#### Locations and seasons

Multi-location evaluations must be carried out throughout the breeding process, with emphasis on the end-user acceptability of resulting advanced lines of the vegetables of interest. During the cultivar development process, decisions on the number and locations of testing sites for evaluation of populations and developed lines should take into account the range of agro-climatic conditions under which the species is cultivated, the existing breeding stations or experimental farms, and the available resources to allocate to such experiments. For example, in West Africa, *G. gynandra* is cultivated in both semi-arid regions (e.g., southern regions of Niger and Burkina Faso, northern Benin, Togo, and Ghana) and sub-humid areas (Central and Southern Benin, Togo and Ghana), under both rain-fed and irrigated cultivation systems. The suite of testing sites should therefore be representative of the target market region, and the selection process should account for region-specific breeding targets; and running multi-year trials would help ensure accurate evaluation of variety performance. Importantly the sites selected should reliably and effectively differentiate lines for their target environments. Many different tools are available to assist breeders throughout the selection process (e.g., GGE Biplot, R package “selectiongain”)^140,141^. These can be used to optimize the selection and testing environments. The Breeding Management System software developed by the Integrated Breeding Platform (www.integratedbreeding.net) is particularly noteworthy, as it facilitates the statistical analysis of genotype x environment interactions, employs mixed model analysis to compute estimates of heritability, and offers Best Linear Unbiased Predictors (BLUPs) and Estimates (BLUEs) to facilitate selection. The platform also serves as an efficient repository for the vast amount of phenotypic, genotypic, and genomic data generated by a breeding program.

#### Agronomic practices pertaining to optimal performance of cultivars

A breeding program seeks to develop new high-yielding cultivars adapted to the range of environments in the target region; therefore, investigating the impact of agronomic practices on desired traits is a critical aspect of multi-environment testing of both breeding lines and products destined for release. Whenever possible, the agronomic practices utilized in testing should reflect existing cropping systems in the market region. More generally, the various impacts of different fertilization schemes (organic or chemical fertilizers, doses and frequencies of applications), soil tillage regimes (tillage vs. no tillage), irrigation practices (doses and frequencies of water supply, waterlogging), planting densities, and  harvesting modes (e.g., rooting, cutting, leaf picking) on growth, regrowth (when applicable), and overall yield are important factors to take into account throughout the breeding process. Once genetically improved varieties are created, a final step before release involves identifying agronomic practices to maximize cultivar performance and productivity.

#### End-user evaluation

The foremost goal of a breeding program is the wide adoption of released cultivars that satisfy the needs of both producers and consumers. End-users should therefore be involved at different stages of the breeding process, including the up-front definition of breeding objectives, the selection of populations or lines with superior characteristics, and the evaluation of final products which also serve as starting points for further breeding efforts.

The outputs of the breeding program should be first presented to target producers for evaluation and selection in coordinated structured trials. The “mother and baby” trial design^142^, which consists of within-site replicates of a set of cultivars/accessions on research stations (mother) and single replicate satellite trials of subsets of cultivars (babies) in larger plots on farmers’ fields, could be adopted as an integrated way to involve farmers, local seed companies, as well as research institutes. Such a design allows elicitation of the evaluation criteria of growers, assessment of the impact of farmer practices on cultivar performance, and development of guidelines for farmer field schools. Consumer preferences may be determined based on tasting panels and/or test cultivars brought to points-of-sale to assess their marketability and storability. End-user preferences may differ according to socio-cultural, ecological, and economic contexts; therefore, these dimensions should be taken into account to define the geographical areas for such exercises.

### Orphan leafy vegetables value chain development

Translating the research efforts for orphan leafy vegetables breeding into concrete outputs for end-users requires the creation of sustainable collaboration frameworks for stakeholders along the value chain. Analysis of constraints and opportunities for the development of these species as commercial crops should involve researchers, farmer organizations, seed companies, traders, policy-makers, and consumers. Outreach activities could include promotional campaigns highlighting the nutritional benefits and commercial opportunities provided by the species, in partnership with restaurants, schools, and the media. Other key components of value chain development for orphan crops include the development of cropping systems with agronomic practices that facilitate maximum productivity; the creation of post-harvest management best practices, addressing such issues as appropriate harvesting time, drying, and packaging methods to ensure optimal shelf-life; and the provision of adequate resources for farmer training. Concerted and coordinated efforts for the improvement of vegetable seed systems by researchers, seed companies, and farmers is needed to ensure the development of standardized seed quality control regulations and the release and distribution of readily available, high-quality seeds. A well established seed production system using established techniques including a controlled seed multiplication system with isolation, genetic and purity checks as well as a seed distribution system is essential. Outreach about the value of improved cultivars is critical through extension and early adopter farmer trials. The importance of good seed stewardship cannot be underscored. To this end, AGRA has created over 114 seed companies through its programs to address these needs in Africa. This includes training of growers to produce high-quality seed.

Ultimately, the ongoing funding of breeding programs should rely on collaboration between local seed companies and other stakeholders involved in orphan leafy vegetables value chain development. Financial and technical support provided by international organizations involved in orphan crop breeding, such as the Consultative Group for International Agricultural Research (CGIAR) and the African Orphan Crops Consortium, among others, is also critical for capacity building and strengthening of local plant breeding capacity.

## Conclusion and the road forward

Large-scale cultivation and increased commercialization of orphan leafy vegetables is desirable given their excellent nutritional properties and economic potential to stabilize nutritional food security in developing countries. Large-scale production cannot, however, be achieved without concerted efforts from stakeholders across the value chain, from research to production to marketing to end-use. This review highlighted the recent efforts for catalysing the systematic breeding and promotion of large-scale cultivation of these species in West and East Africa. The synthesis emphasized the need for (i) raising awareness of the potential of orphan leafy vegetables to contribute to food and nutritional security in Africa; (ii) increased and coordinated germplasm collection and characterization of the species; (iii) investigation of the genetic, physiological, biochemical, and molecular processes underlying key traits of interest; (iv) traditional genetics and genome-enabled research targeting trait development; (v) breeding efforts taking advantage of the advances in “omics” disciplines and the available comparative resources in related species; and (vi) expanded collaboration among local researchers, value chain stakeholders, and international organizations interested in orphan crops to sustain technical and financial support for orphan crops breeding programs.

## Electronic supplementary material

Supplementary File 1 *Gynandropsis gynandra* specimen collection form

Supplementary File 2 Morphological descriptors of *Gynandropsis gynandra*
