# Supplementary material for: A roadmap for breeding orphan leafy vegetable species: a case study of Gynandropsis gynandra (Cleomaceae)
Source: Hortic Res. 2018 Jan 10;5:2. doi: 10.1038/s41438-017-0001-2 (PMC5798814; doi:10.1038/s41438-017-0001-2)
Supplement: Supplementary file 1 — Gynandropsis gynandra specimen collection form [file 41438_2017_1_MOESM1_ESM.pdf]

Gynandropsis gynandra specimen collection form

Date (dd-mm-yy)		Collection No:		Part(s)and amount collected	
Collectors:				Photo No(s).	
Collecting institute(s)				Country:	
Region/ County		Ward or location		Village:	
Site description (e.g name, distance and direction from nearest town)					
Latitude:		Longitude		Altitude (M)	
Local name of plant & Language				Variety/ type	
Seed source					
Plant description: growth form e.g. erect, spreading, branching; height, hairiness, petal colour, fruit, leaves, other observations e.g. pests and diseases					
Germination type: (Direct seeding, spontaneous/ tolerated, transplanting)		Frequency/ abundance (e.g rare, occasional, abundant)			
Topography of landscape (flatland, valley bottom, hill/ridge side, hill/ridge top, undulating land)		Habitat e.g. Kitchen garden, main farm, bushland, wooded grassland, swamp, riverine, other			
Gradient/ slope (%)		Soil type – colour, texture (sandy, loamy, clay, rocky)			
Associated plant species (local, English, French or scientific names)					
Plant uses e.g. food, income, medicine.					
Other observations (e.g. cultural beliefs; agronomic & management practices-irrigation, monoculture, mixed; harvesting type, frequency, inputs, disease and pest management; processing; horticultural traits e.g. water stress tolerance, pest resistance, yield, seed selection, storage; domestication; nutritional traits/ health benefits					
Contact details of respondent (mobile number					

Utilizing the genome of the vegetable species *Cleome gynandra* for the development of improved cultivars for the West and East African markets
