# Supplementary material for: A roadmap for breeding orphan leafy vegetable species: a case study of Gynandropsis gynandra (Cleomaceae)
Source: Hortic Res. 2018 Jan 10;5:2. doi: 10.1038/s41438-017-0001-2 (PMC5798814; doi:10.1038/s41438-017-0001-2)
Supplement: Supplementary file 2 — Morphological descriptors of Gynandropsis gynandra [file 41438_2017_1_MOESM2_ESM.pdf]

Utilizing the genome of the vegetable species *Cleome gynandra* for the development of improved cultivars for the West and East African markets

CHARACTERIZATION RECORD SHEET

(Descriptors for *Gynandropsis gynandra*)

Crop/ species:	***Cleome gynandra* [*Gynandropsis gynandra*]**		
Characterization by:	………………………	Date:	………………………
Cultivated/spontaneous:	………………………	Location/ site:	………………………
Plot No. :	………………………	Accession No. :	………………………
Sowing Date:	………………………	Other plant name:	………………………
Transplanting Date:	………………………	Age (weeks):	………………………
Origin of seed:	………………………		………………………

SEEDLING DATA

1. **Germination period** ______
   (no. of days from sowing to first germination)

2. **Anthocyanin coloration of hypocotyl** ______
   (when the seedling primary leaves are fully opened and the terminal bud is around 5 mm)
   0 = Absent 1 = Present X = Mixture

3. **Hypocotyl color intensity** ______
   3 = Light 5 = Medium 7 = Dense X = Mixture

VEGETATIVE DATA

4. **Plant height** (cm) (N=10) ______
   (to be measured at the same time for all the accessions 6 to 10 weeks after sowing)
   ______ ______ ______ ______ ______ ______ ______ ______ ______ ______

5. **Stem diameter at the first branch** (mm) (N=10) ______
   ______ ______ ______ ______ ______ ______ ______ ______ ______ ______

6. **Growth habit** ______
   3 = Upright *(erect narrow crown)* 5 = Intermediate 7 = Spreading *(wide)* 7 = Prostrate X = Mixture

7. **Branching habit** ______

0 = None 3 = Sparse (<= 5) 5 = Intermediate (6-10) 7 = Abundant (> 10)
X = Mixture

8. **Color of main stem** *(Scale from 0=entirely green to 5= entirely purple)* (N=10) ______

______ ______ ______ ______ ______ ______ ______ ______ ______ ______

9. **Stem hairiness** ______
3 = Glabrous 5 =Scantly hairy 7 = Moderately hairy 9 = Very hairy or woolly

10. **Leaf blade leaflets** *(dominant number of leaflets per leaf, excluding inflorescence)* ______
3 = Very low (3 leaflets) 5 = Low (4 leaflets) 7 = Intermediate (5 leaflets)
9 = High (6 leaflets) 11= Very high (7 leaflets) X = Mixture

11. **Leaf color** ______
3 = Light green 5 = Green 7 = Dark green X = Mixture

12. **Terminal leaflet shape** ______
3 = Elliptic 5 = Ovate 7 = Lanceolate 9 = Obovate 11 = Oblanceolate
13 = Deltoid 15 = Spatulate X = Mixture

13. **Terminal leaflet length** (cm) (N=10)* ______

______ ______ ______ ______ ______ ______ ______ ______ ______ ______

14. **Terminal leaflet width** (cm) (N=10)* ______

______ ______ ______ ______ ______ ______ ______ ______ ______ ______

15. **Petiole length** (cm) (N=10)* ______

______ ______ ______ ______ ______ ______ ______ ______ ______ ______

16. **Leaf area** (cm^2) (N=10)* ______

______ ______ ______ ______ ______ ______ ______ ______ ______ ______

17. **Leaf pubescence/ hairiness on upper surface** ______
3 = Not hairy/scabrous 5 = Sparsely hairy 7 = Hairy 9 = Densely hairy
X = Mixture

18. **Color of petiole** ______
1 = Green 3 = Green tinged purple 5 = Purple tinged green 7 = Purple

19. **Oiliness / wax level on leaves and stems** ______
3 = None 5 = Fairly waxy 7 = Very oily/sticky

20. **Upper surface veins** ______
3 = Not depressed 5 = Slightly depressed 7 = Highly depressed

INFLORESCENCE DATA

21. **Flowering date** (N=10) ______

______ ______ ______ ______ ______ ______ ______ ______ ______ _

22. **Flower color** (when fully opened) ______

1 = White 2 = Yellow 3 = Orange
4 = Red 5 = Rose-red 6 = Pink
7 = Purple 8 = Cream X = Mixture

23. **Length of filament** (cm) (N=10) ______

______ ______ ______ ______ ______ ______ ______ ______ ______ ______

24. **Length of gynophore** (mm) (N=10) ______

______ ______ ______ ______ ______ ______ ______ ______ ______ ______

25. **Length of androphore** (mm) (N=10) ______

______ ______ ______ ______ ______ ______ ______ ______ ______ ______

26. **Length of pedicel** (mm) (N=10) ______

______ ______ ______ ______ ______ ______ ______ ______ ______ ______

FRUIT & SEED DATA

First seed harvest date _______________

Last seed harvest date _______________

27. **Days to fruiting** (no. of days from sowing to mature pods) (N=10) ______

______ ______ ______ ______ ______ ______ ______ ______ ______ ______

28. **Pod length** (mm) (N=10) ______

______ ______ ______ ______ ______ ______ ______ ______ ______ ______

29. **Pod width** (mm) (N=10) ______

______ ______ ______ ______ ______ ______ ______ ______ ______ ______

30. **Immature pod color** ______

1 = Green 3 = Green purple 5 = Light purple 7 = Purple

31. **Mature pod color (not dry, bearing mature seeds)** ______

3 = Green 5 = Yellow-green 7 = Yellow 9 = Brown X = Other

32. **Shape of cross-section of mature or nearly mature fruit** ______

3 = Rounded, 5 = Elliptic/oblong

33. **Pod surface** ______

3 = Shiny smooth 5 = Slightly rough 7 = Scabrid (v. rough)

34. **Pod shape** ______

1 = No folds (regular) 3 = With few folds 5 = With many folds

35. **Color of mature healthy seeds** ______

3 = Black 5 = Grey 7 = Other

36. **1000-seeds weight (mg)** ______

Weigh 8 samples of 100 seeds separately- the coefficient of variation should be inferior to 4 in order to consider the average weight – multiply the average weight by 10

______ ______ ______ ______ ______ ______ ______ ______

YIELD DATA

37. **Total biomass (g)** ______

______ ______ ______ ______ ______ ______ ______ ______ ______ ______

38. **Edible biomass (g)** ______

______ ______ ______ ______ ______ ______ ______ ______ ______ ______

39. **Leaf dry matter content (%)** ______

______ ______ ______ ______ ______ ______ ______ ______ ______ ______

Remarks (Observation of insect pests or diseases/special forms of flowers etc.)
